# Mandibular Ramus: An Indicator for Gender Determination

**DOI:** 10.7759/cureus.34192

**Published:** 2023-01-25

**Authors:** Pradnya Harish Karmarkar, Amit Mhapuskar, Darshan R Prasad Hiremutt, Isha Prasad Kale, Meenal Tepan, Prashant Rao

**Affiliations:** 1 Department of Oral Medicine and Radiology, Bharati Vidyapeeth (Deemed to be University) Dental College and Hospital, Pune, IND; 2 Department of Oral Pathology and Microbiology, Bharati Vidyapeeth (Deemed to be University) Dental College and Hospital, Pune, IND

**Keywords:** gonial angle, gender determination, mandibular ramus, sexual dimorphism, orthopantomograph, digital opg, forensic odontology

## Abstract

Background

The goal of this study is to use digital orthopantomographs (OPGs) to find out the role the mandibular ramus plays in figuring out a person's gender.

Methodology

Six hundred digital OPGs of patients, aged 21 to 50 years of either gender, fulfilling the exclusion and inclusion criteria, were randomly selected from the department archives exclusively for this digital retrospective study. All the scans were anonymized before the analysis. A total of seven measurements (in mm) were performed on OPGs, namely, minimum and maximum widths of the ramus, minimum and maximum condylar heights, maximum height of the ramus and coronoid, gonial angle bilaterally, and bigonial width. The data obtained was statistically analyzed using IBM SPSS Statistics for Windows, Version 21.0. (IBM Corp., Armonk, NY, USA), by a stepwise discriminant functional analysis for gender determination.

Results

Linear measurements, including maximum and minimum widths of the ramus, maximum height of the condyle, height of the ramus, and coronoid and bigonial width, revealed more values in males than in females. However, the gonial angle showed higher average values in females than in males. Moreover, all seven parameters showed statistically insignificant age-related changes.

Conclusions

The mandibular ramus showed high sexual dimorphism, and its analysis on OPG can be a valuable aid in the determination of gender in the fields of forensic odontology and anthropology.

## Introduction

The skull is the most dimorphic and easily sexed portion of the skeleton after the pelvis, providing an accuracy of up to 92%. However, in cases where an intact skull is not found, the mandible may play a vital role in gender estimation as it is the most dimorphic, largest, and strongest bone of the skull [1]. Due to the presence of a dense layer of compact bone, it becomes a very durable and well-preserved bone than many other bones. Mandibular ramus can be used to differentiate between sexes, and it also expresses strong univariate sexual dimorphism [2].

Extra-oral digital dental radiography is becoming more popular in routine dental practice [3]. The ease of storing digital radiographic images and reproducing them when required has made digital radiography an important tool in forensic anthropology. Several radiographic images act as an aid for utilization in age and gender discerning, which includes panoramic radiographs, lateral oblique X-rays, digital imaging, lateral cephalograms, and advanced imaging technologies such as CT, MRI, and cone beam CT (CBCT) [4]. Among these, orthopantomographs (OPGs) are the most common extra-oral radiographs for viewing the maxillo-mandibular structures in dentistry [5]. As there are no overlying bony structures above the mandible, so it could be utilized as a pivotal aid for radiological gender identification [6]. When skeleton sex determination is considered, metric analyses on the radiographs are often found to be of superior value owing to their objectivity, accuracy, and reproducibility [1]. In forensic anthropology, a calibrated measurement tool was used and antemortem radiographs provide the cornerstone of the identification of human remains. The presence of plenty of panoramic radiographs provides a great opportunity to study sexual dimorphism and age estimation of individuals in certain populations [7]. The sex of an unknown individual can be determined based on the data from the morphology and metric features of the skull and the mandible, soft tissues, dental records, as well as by DNA analysis of teeth [1].

Gender recognition is necessary for forensic science. Literature reveals a discrepancy in results as Humphrey et al. [8] reported that females have a significantly larger gonial angle as compared to males, whereas Al-Faleh [9] and Raustia and Salonen [10] were not able to determine any significant disparity between genders and gonial angle. In another study by More et al. [11], the correlation of gender with the morphology of mandibular ramus was significant (*P* < 0.05), with the overall accuracy for diagnosing sex being 69%, whereas for diagnosing male and female, the accuracy was 68% and 70%, respectively. Antemortem orthopantomograms could also be a valuable tool in recognition as they are commonly accessible and are utilized in daily clinical practice to appraise mandibular fundamental structures bilaterally. Thus, the presence of plenty of digital panoramic X-rays provides a great opportunity to study sexual dimorphism as it covers maxillomandibular structures completely [2].

It is a well-known fact that the development and growth of the mandible show variation between the two genders [12]. Hence, it is imperative to study the ramus of the mandible and gonial angle for gender discernment.

## Materials and methods

This study was a digital retrospective study. For this study, 600 digital OPGs of patients, both male and female aged 21 to 50 years, fulfilling the exclusion and inclusion criteria, were randomly selected from the archives of the department of oral medicine and radiology. The scans were anonymized before the evaluation of the selected parameters. The scans included in the study were of completely dentulous mandibles with proper positioning. The scans that were not included in the study were those where gross deformity of the maxilla-mandibular structures could be seen with any artifacts on the radiograph, any lesions in the mandibular arch that were radiolucent or radiopaque, premolars that were missing, mixed dentition, trauma history and/or ongoing or completed trauma treatment, and radiographic evidence of temporomandibular joint (TMJ) problems. There were two providers involved in collecting the measurements. One was a subject expert with more than 20 years of experience with OPG and its interpretations. The other was a postgraduate student with more than one year of experience in OPG and its interpretation.

A pilot study of 30 scans was done by both observers. The interobserver reliability was assessed through kappa statistics for the degree of agreement. From the overall kappa statistics value, we found out that an almost perfect agreement was achieved between the two providers overcoming any chance of bias and good generalizability. Calibration was then done for both observers, and then the remaining research was completed. Also to eliminate the measurement and provider bias, the analysis of the same anonymized scan was done by both observers twice each with a gap of 15 days.

A total of seven measurements (in mm) were performed on the digital radiographic image (Table 1).

**Table 1:** Measurements performed on the digital radiographic image.

A: Maximum width of ramus: It is the greatest anteroposterior diameter of the ramus.
B: Ramus minimum width: It is the smallest anteroposterior diameter of the ramus.
C: Maximum height of condyle: Beginning at the uppermost point on the condyle of the mandible to the lower point of the mandible.
D: Maximum ramus height: The distance between the mandibular condyle's uppermost projection point and the bone's lower border
E: Maximum height of coronoid: The projective distance between the coronoid and the lowest point of the bone. It is a tangential outline that goes from the lowest points at the gonial angle and the lower border of the mandibular body to the posterior borders of the condyle and ramus.
F: The point of intersection of these two outlines led to the formation of the gonial angle.
G: Bigonial width: This is the horizontal area between two gonia, which is calculated between the right and left gonia

**Figure 1:**
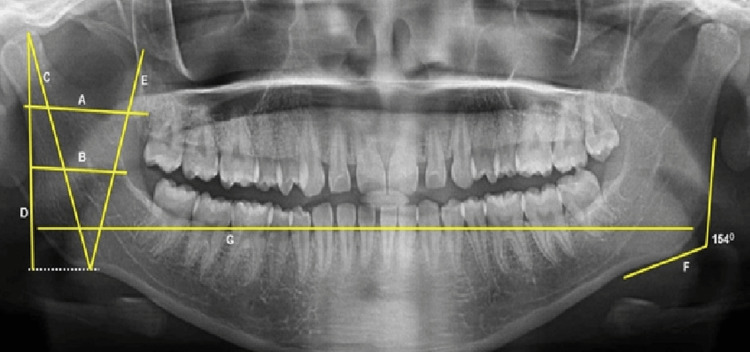
Image showing OPG measurements. OPG, orthopantomograph

The method of analysis of various parameters was as follows on an OPG (Figure 1).

These parameters were most stable and easy to identify on the mandible. The most convex point on the inferior border of the mandible in the posterior region is the lowest point on the bone. Hence, they were chosen for this study, which had a large sample size of 600 OPGs, after consulting a biostatistician and considering previous studies. These parameters were also considered in a previous study by Indira et al. [2] and Mostafa and El-Fotouh [13] that had a smaller sample size (<100).

The measurements were noted in tabular form. Each gender group was then split into three groups based on age to find out how the parameters of the mandible changed with age. The obtained data for categorical variables was shown as *n*% of cases, while the data for continuous variables was shown as mean and standard deviation (SD). The statistical intergroup comparison was estimated using the independent sample *t-*test or unpaired *t*-test. The statistical comparison of the intergroup distribution of means of continuous variables was also tested using the analysis of variance (ANOVA) procedure. For paired comparisons of means of continuous variables, the paired *t*-test was used. The linear discriminant function analysis was carried out to obtain a linear combination of various measurements that characterize the two classes of gender. Any underlying normality assumption was evaluated before applying the study variables for the *t*-test and ANOVA, and *P*-values < 0.05 were considered to be statistically significant. IBM SPSS Statistics for Windows, Version 21.0. (IBM Corp., Armonk, NY, USA) was used to figure out gender in the statistical analysis.

## Results

The means of all the parameters on the left and right sides as well as their combined means within the age category (21-50 years) are illustrated in Tables 2-3. We found that all seven parameters show a statistically significant difference between the two genders.

**Table 2:** The linear discriminant function based on various mandibular ramus measurements for each sex (all age groups combined). Max, maximum; Min, minimum

Variable	Right side		Left side		Combined	
	Male	Female	Male	Female	Male	Female
Constant	-9214.64	-7656.54	-8878.17	-7285.49	-16408.50	-14437.30
Max. ramus width	57.24	54.65	9.96	11.81	16.35	16.80
Min. ramus width	-37.37	-34.19	-27.30	-24.54	-30.53	-26.13
Max. condylar height	29.37	26.69	30.28	27.58	57.29	52.93
Max. ramus height	40.03	34.82	43.95	37.04	19.19	12.31
Max. coronoid height	34.97	38.14	24.92	27.61	36.93	40.32
Angle	58.49	66.29	65.73	70.65	64.84	54.72
Bigoneal width	-	-	-	-	84.44	84.56

**Table 3:** The distribution of accuracy of prediction of sex based on various mandibular ramus measurements (combined measurements).

True group	Predicted group membership		Total	Accuracy (%)
	Male	Female		
Male	268	32	300	89.67
Female	30	270	300	
Total	298	302	600	

The mean bigonial width in males was 182.5 mm, while it was 176.79 mm in females. The mean bigonial angle in males was 154.73°, while in females, it was 172.45°. The mean values of the maximum ramus width (31.09 mm), minimum ramus width (29.30 mm), maximum condylar height (57.38 mm), maximum ramus height (66.46 mm), and maximum coronoid height (56.66 mm) were greater in males compared with the values of the maximum ramus width (30.45 mm), minimum ramus width (28.73 mm), maximum condylar height (52.12 mm), maximum ramus height (60.25 mm), and maximum coronoid height (55.90 mm) in females. There was no statistically significant difference in the values of the selected parameters by age group (Table 4).

**Table 4:** The comparison of various mandibular ramus right-sided measurements between male and female subjects studied (overall age group). Min, minimum; Max, maximum; SD, standard deviation

Parameters	Right side (*n* = 600)					
	Male (*n* = 300)		Female (*n* = 300)		*T*-value	*P*-value
	Mean	SD	Mean	SD		
Max. ramus width	31.20	0.72	30.34	0.94	12.57	0.001
Min. ramus width	29.66	0.79	28.68	1.47	10.18	0.001
Max. condylar height	57.41	2.18	51.55	1.82	35.73	0.001
Max. ramus height	66.47	0.55	60.31	1.22	79.89	0.001
Max. coronoid height	56.63	0.77	55.96	1.04	8.99	0.001
Angle	154.28	2.17	172.32	0.47	140.71	0.001

A discriminant analysis was performed to predict the gender and statistically significant results were reported with predicted group accuracy of 89.67%. This shows that the mandible shows a high degree of sexual dimorphism and that these parameters can be used to determine the gender of an individual, as required in forensic medicine, using digital OPG.

## Discussion

In cases of accidental injuries, the mandible can survive in a well-conserved state for much longer than any other bone. Hence, it is considered one of the strongest bones in the human skeleton and can be used for antemortem and postmortem forensic analysis [6,14]. In this study, a total of seven measurements (in mm), namely, the maximum and minimum width of the ramus; maximum height of condyle, ramus, and coronoid; gonial angle; and bigonial width revealed statistically significant differences between the two genders. Hence, this methodology used for gender determination was useful for creating a logistic regression model using various parameters that presented 89.67% accuracy in gender estimation, which can be useful in forensic sciences. Garg et al. [15] conducted a retrospective study analysis by using 180 digital OPGs (60 from each ethnicity: 60 Indians, 60 Malays, and 60 Chinese) and reported that the maximum ramus breadth has demonstrated the greatest difference in the mean values, which are lowest in Chinese, followed by Malays and Indians, and reported a statistically significant correlation between the maximum ramus breadth with ethnicity with *P *= 0.008. Another similar study by Loth and Henneberg [16] proved that the mandibular ramus flexure plays an important role in the determination of gender up to an accuracy of 94% to 99% in combined African and American samples. Our results are comparable to the results of a study carried out by Indira et al. [2], in which it was revealed that the mandibular ramus can be employed to distinguish between genders as well as express strong univariate sexual dimorphism. Saini et al. [17] conducted a study on dry adult mandibles from the northern part of India and found that the ramus expresses strong sexual dimorphism in this population with an overall accuracy of 80.2%. Moreover, the coronoid height was the single best parameter with an accuracy of 74.1%. Another study by Pokhrel and Bhatnagar [18] reported an accuracy of 70.9% to 82.9%. This study reported predicted group accuracy of 89.67%. Saloni et al. [19] reported that the overall accuracy for determining sex from mandibular ramus was found to be 77.6%, whereas for determining male or female, the accuracy was 78.4% and 76.8%, respectively.

In this study, in males, the mean values of the maximum ramus width, minimum ramus width, maximum condylar height, and maximum ramus height were greater, while in females, the values of the maximum ramus width, minimum ramus width, maximum condylar height, maximum ramus height, and maximum coronoid height were lower. The results of this study match the results of the studies by Indira et al. [2], Saini et al. [17], and Damera et al. [1]. This study found that the mean bigonial width in males was 182.5 mm, while it was 176.79 mm in females. Furthermore, the mean bigonial angle in males was 154.73°, while in females, it was 172.45°. Similar to our results, the gonial angle is positioned at the posterior border of the intersection of the inferior border of the mandibular ramus. In all racial groups, the mean angle in females is 18° higher than it is in males [12,20]. Hence, similar results were found in this study of 600 OPG scans.

Generally, the gender can be accurately identified after completion of the development of the concerned bone, thus justifying this study's age group selection of 21 to 50 years. However, this study model cannot be used for age estimation, as it agrees with other similar studies by Mehta et al. [14] and Shah et al. [20]. This shows that the mandible shows a high degree of sexual dimorphism and that these parameters can be used to determine the gender of an individual, as required in forensic medicine, using digital OPG. Among various anatomical landmarks in the mandible, the gonial angle and ramus of the mandible are regarded as stable landmarks [6,7,21]. It is believed that the mandibular ramus differentiates between genders due to the differences in the stage of development, growth duration, and growth rates [6]. The characteristics of skeletal structures differ in various populations, emphasizing the requirement for population-specific osteometric criteria as well as standards for the determination of gender. Also, many studies have been carried out in India on gender determination using mandibular ramus, but most of them have had a sample size of fewer than 100 OPGs [14]. Hence, a sample size of 600 was used to plan this study, which gave reliable results.

The comparative analysis of postmortem and antemortem radiographs is one of the keystones in the confirmed identification of human skeletal remains in forensic anthropology. In addition, evaluation of the demarking point reveals an important role in identifying the gender when a single variable is available, as in incomplete or mutilated mandibles. It can be observed from the means of the variables that the minimum and maximum ranges in males were higher than those in females. Hence, statistically, one can find whether the provided sample is of a male or a female by evaluating it with the stated dimension and referring to the demarking point [22]. The results of this study revealed that gender estimation can be successfully carried out in forensic odontology by using various parameters on the mandibular ramus and gonial angle, which is linear to studies done previously with a smaller sample size as well as a population belonging to a specific geographical region [23,24]. The results of the study are in agreement with previous gender estimation studies done using other imaging modalities like CBCT, lateral cephalogram, etc. [25].

The limitation of the study was the small sample size and inclusion of a very small area for gender estimation. This study should have incorporated and compared other techniques for gender determination.

## Conclusions

Forensic odontology, using various methods, is helpful in determining gender, age, etc. The specific geographical condition and its fusion with techniques of forensic evaluation can greatly help in the determination of various parameters. Gender estimation can be successfully carried out in forensic odontology using various parameters on the mandibular ramus and gonial angle. The mandible is resistant to damage and the disintegration process. Hence, we conclude that the use of the ramus of the mandible and gonial angle is recognized as a support for determining gender in forensic anthropology.
